# Malaria prophylaxis policy for travellers from Europe to the Indian Sub Continent

**DOI:** 10.1186/1475-2875-5-7

**Published:** 2006-02-01

**Authors:** RH Behrens, Z Bisoffi, A Björkman, J Gascon, C Hatz, T Jelinek, F Legros, N Mühlberger, TropNetEurop, P Voltersvik

**Affiliations:** 1 Hospital for Tropical Diseases & London School of Hygiene & Tropical Medicine, London, UK; 2 Centro per le Malattie Tropicali, Ospedale S. Cuore, Negrar Verona, Italy; 3 Department of Medicine, Unit of Infectious Diseases, Karolinska Institute, Stockholm, Sweden; 4 Centre de Salut Internacional, Hospital Clinic, IDIBAPS, Barcelona, Spain; 5 Swiss Tropical Institute, Basel, Switzerland; 6 Centre National de Référence pour le Paludisme d'Importation et Autochtone, France; 7 Institute of Tropical Medicine, Berlin, Germany; 8 The Gade Institute, Haukeland University Hospital, Bergen, Norway

## Abstract

Analysis of malaria imported into eight European countries from the Indian sub-continent (ISC) (India, Pakistan, Bangladesh and Sri Lanka) led to a consensus statement on the use of chemoprophylaxis within TropNetEurop. The proportion of cases from the ISC in 2004 ranged from 1.4%–4.6% of total imported cases. *Plasmodium falciparum *cases reported from the eight countries was only 23 (13% of all cases from the region). Total malaria reports between 1999–2004 fell from 317 to 180. The risk of malaria in UK residents visiting the region was > 1 case per 1,000 years exposed. The group recommended non-selective prescribing of chemoprophylaxis for visitors to India, Pakistan, Bangladesh and Sri Lanka should be dropped.

## Introduction

TropNetEurop is an electronic network which links surveillance data on imported infectious diseases throughout Europe. The network is designed to identify emerging trends in regional or global destinations as they affect European travellers. Sentinel surveillance is carried out by participating clinical sites using rapid case reporting. This data can serve as a convenient tool to identify changes relevant to Public Health bodies and focus further investigations.

A number of countries in the Indian Subcontinent (ISC) including India, Pakistan, Bangladesh and Sri Lanka continue to report endemic malaria transmission to the World Health Organisation [1]. Malaria prevention in travellers from most European and North American countries to this region has relied on the use of chemoprophylactic drugs. Although *Plasmodium vivax *malaria has been the most frequent species in surveillance reports, ongoing transmission of *Plasmodium falciparum *has meant that the recommended drug regimens have included drugs to prevent both falciparum and vivax malaria. The number of imported cases of malaria reported from these countries has been noted to be declining and the continued use of chemoprophylaxis for travellers to these countries has been questioned [2]. Switzerland and Germany have discontinued recommending chemoprophylaxis and introduced standby therapy for travellers to the ISC [3].

## Discussion

In Sweden, routine chemoprophylaxis for travellers to India is not recommended [4].

Reports to National malaria surveillance bodies of malaria cases contracted in the above four countries between 1999 and 2004 were analysed. The data was provided through TropNetEurop members who have access to national data and who could provide details by year and, where possible, species. The data from the UK was complemented by data from the International Passenger Survey (IPS). The IPS is a year round survey of incoming and outgoing passengers at all major exit ports. Around 0.2% of all travellers are interviewed and this sample provides estimates of the total annual visits (and their duration of stay) made by UK residents to individual countries worldwide. The data from France provided through a reporting network of 120 selected hospital laboratories covers approximately half of annual estimates of malaria cases to the National Reference Centre for Imported and Autochtonous Malaria Epidemiology (CNREPIA).

The attached table details total cases of malaria acquired in the ISC by year and species reported to surveillance bodies of individual European countries (Table 1). Malaria cases from India make up a very small proportion of the total reports (range 1.5%–4.6%). The number of cases of imported *P. falciparum *from the ISC in 2004 from reporting countries was 25 of a total of 118 cases. The total numbers of imported malaria from the 8 countries over the past three years has been less than 200 per year.

**Table 1:** Table shows all cases of all species malaria and cases of *Plasmodium falciparum *(in brackets) from the ISC, as a proportion of total malaria reports in 2004. UK data represents cases occurring in UK residents only and not visitors or immigrants.

	**1999**	**2000**	**2001**	**2002**	**2003**	**2004**	**ISC % of total malaria reports**
**Switzerland**							3.50%
India	16(2)	6	7(2)	6(1)	6	4(1)	
Pakistan	0	1	1	0	1	1	
Bangladesh	0	0	0	0	0	0	
Sri Lanka	5	1	1	1	2(1)	0	
**United Kingdom**							3.50%
India	40(7)	32(5)	25(3)	22(2)	22(1)	29(2)	
Pakistan	43(3)	57(1)	50(2)	40(1)	28	38(2)	
Bangladesh	0	0	0	1	1	0	
Sri Lanka	13(1)	8(2)	12(4)	4(1)	0	1(1)	
**Germany**							1.5%
India			3(1)	6(2)	5(3)	3	
Pakistan			0	2(1)	1	0	
Bangladesh			1(1)	0	0	0	
Sri Lanka			0	0	0	1	
**France**							1.20%
India	19(2)	17(7)	13 (3)	10 (2)	11 (1)	25 (5)	
Pakistan	6(2)	6(1)	10 (3)	12 (1)	8 (1)	4 (0)	
Bangldesh	1 (0)	1(1)	0	0	1 (0)	1 (1)	
Sri Lanka	2 (1)	0	1 (0)	1 (0)	0	3 (0)	
**Sweden**							4.60%
India	7	5(2)	2(1)	4	1	2	
Pakistan	1	0	3	1	2	1	
Sri Lanka	3	0	0	0	0	0	
Bangladesh	0	0	0	0	0	0	
**Norway**							4.17%
India	3		1	3	0	0	
Pakistan	4(1)	5	4(2)	1	2	2(1)	
Sri Lanka	3	1	1	0	0	0	
Bangladesh	1	0	0	0	0	0	
**Italy**							3.00%
India		9(4)	9(1)	5	3	3(1)	
Pakistan			1(1)	1	1		
Bangladesh		1	0	0	0	0	
Sri Lanka		0	1		0	1	
**Spain**							1.53%
India	6(1)	0	2	4(1)	2	6	
Pakistan	1	0	0	0	1	1	
Bangladesh	0	0	0	0	0	0	
Sri Lanka	0	0	0	0	1	0	
TropNetEurop*							1.6%
India	14(4)	26(5)	15(6)	14(4)	14(3)	14(2)	
Pakistan	5	13(3)	19(4)	11(1)	20(2)	15(2)	
Bangladesh	1	1	0	0	0	0	
Sri Lanka			1(1)		1		

* Cases in TropNetEurop data, may be included in national data sets.

In 2004, UK residents made a total of 1.2 million visits to the ISC, staying on average 36 (95%CI 27–46) days per visit. Around 75% of these were made to India and Pakistan. These two countries contributed over 95% of the malaria cases. Analysing all species malaria from these two countries in 2004, the rate in India (stay 31 days, one case per 1,923 years exposed) is lower than in Pakistan (stay 41 days, one case per 1,059 years exposed). The *P falciparum *rates are one case per 27,888 in India and 20,112 years exposed in Pakistan. The rates in Sri Lanka and Bangladesh are much lower than these. In France during 2004, the number of departing travellers to India was 200,000, to Pakistan 12,000, to Sri Lanka 38,000 and to Bangladesh 2,500 This gives an estimated attack rate for malaria of respectively, 0.01%, 0.03%, 0.008% and 0.04% per visitor.

One factor which might explain low infection rates is that most travellers are protected through using chemoprophylaxis. This is not supported in a departure lounge survey at major European airports, which found only 22% of travellers to low risk malaria regions carried anti-malaria drugs[5]. Although the number of visits made from most other countries is unknown, the numbers of imported malaria cases reported in countries providing data is often lower than numbers reported from the UK.

The group consensus was that the likelihood of severe adverse events (leading to stopping prophylaxis) of a number of the regularly used prophylactic agents mefloquine, chloroquine and proguanil or doxycycline (2–5%) [6] are significantly higher than the risk of acquiring malaria in these four countries. Death as a potential severe outcome needs to be considered. In a series of 618 cases P. *vivax *malaria reported to TropNetEurop 1999–2003, 17% were from the ISC and no deaths were reported [7].

## Conclusion

In terms of public health policy, the costs and benefits of prescribing chemoprophylaxis with potential side effects to large numbers of individuals to prevent small numbers of non severe malaria cases appears overwhelmingly unfavourable. Although the risk may vary by region and country within the ISC, this variation is unlikely to be significantly different given the numbers of cases from the continent as a whole. The group proposed that the recommendation of non-selective prescribing of chemoprophylaxis for visitors to India, Pakistan, Bangladesh and Sri Lanka should be dropped.

There was complete consensus on continued recommendation of personal protective measures against biting insects. There was no consensus on provision of standby treatment to travellers to this region. There were national differences and limited agreement on appropriate regimens, target groups and duration of journey for which standby treatment is indicated.

The collaborative analysis of national data enhances epidemiological data quality and allows evidence based policy to be generated and standardised across Europe. This type of analysis needs to be ongoing to identify trends, outbreaks or epidemics. Recognising changes in malaria transmission early is critical to re-adjusting policy and assuring optimal protection of travellers. Careful monitoring of cases acquired from this region after a change in policy will be necessary to detect changing malaria epidemiology amongst travellers.

## Authors' contributions

RB conceived, designed and co-ordinated the study. TJ organised and chaired the policy group meeting. RB, AB, JG, CH, FL, NM and PV obtained and prepared data and contributed to policy and manuscript preparation. All authors read and approved the final manuscript.
